# Generative Adversarial Network of Industrial Positron Images on Memory Module

**DOI:** 10.3390/e24060793

**Published:** 2022-06-07

**Authors:** Mingwei Zhu, Min Zhao, Min Yao, Ruipeng Guo

**Affiliations:** College of Automation Engineering, Nanjing University of Aeronautics and Astronautics, Nanjing 211106, China; zhumingwei74@gmail.com (M.Z.); lilynuaa92@gmail.com (M.Y.); rpguo@nuaa.edu.cn (R.G.)

**Keywords:** attention mechanism, generative adversarial networks, image generation, positron images

## Abstract

PET (Positron Emission Computed Tomography) imaging is a challenge due to the ill-posed nature and the low data of photo response lines. Generative adversarial networks have been widely used in computer vision and made great success recently. In our paper, we trained an adversarial model to improve the industrial positron images quality based on the attention mechanism. The innovation of the proposed method is that we build a memory module that focuses on the contribution of feature details to interested parts of images. We use an encoder to get the hidden vectors from a basic dataset as the prior knowledge and train the nets jointly. We evaluate the quality of the simulation positron images by MS-SSIM and PSNR. At the same time, the real industrial positron images also show a good visual effect.

## 1. Introduction

In recent years, the Generative adversarial network (GAN) [1] has been the state-of-the-art image generation model since it was proposed in 2014. The original GAN consists of a generative model G and a discriminative model D. The two models are coupled tightly and trained simultaneously. While the generative model is trained to get data G(z) from random noise z, the discriminative model is trained to discriminate between real and generated data. The whole model is constantly optimized during the training. GANs have been used in many applications and most of them have achieved great performance. Such as image generation [2], single image super-resolution [3], image style transfer [4], and image inpainting [5].

Many network structures have been presented based on the original GAN. Ref. [6] used a CNN (Convolution Neural Network) to establish the framework and training mode of GAN. Ref. [7] proposed Wasserstein GAN (WGAN) to measure the distance between generated and real data by Earth-Mover and largely solved “pattern collapse”. Ref. [8] proposed Conditional Generative Adversarial Nets (CGAN) and introduced constraints to improve the stability of sample generation. Ref. [9] of the NVIDIA team proposed a progressive structure model to realize the transition from a low-resolution to a high-resolution image, and the generative model of the high-definition image can be trained smoothly.

Positron Emission Computed Tomography (PET) is a highly sensitive functional imaging technology. Compared with other traditional industrial non-destructive testing methods, such as X-rays and CTs, the gamma photons produced in the positron annihilation process have a stronger penetrability and a lower radiation. Therefore, it has a good application prospect in high-precision industrial closed cavity detection.

Under the current conditions, the number of industrial samples is low. Due to the ill-posed nature of the inverse problem, high noise and artifacts inevitably exist in the final imaging results, which affect the image quality. Therefore, we have to improve the quality of the reconstructed images in order to facilitate further defect handing and fault troubleshooting.

Considering the following two problems of industrial positron images: data scare and poor quality. In this paper, we propose a positron image adversarial model based on the attention mechanism. Firstly, we use medical images (an open-source dataset from NIHCC) to train a basic generative network. Then, the memory module is built based on the contribution of the details of the positron image to the image quality. Finally, we aim to build an adversarial network that focuses on industrial positron images.

In summary, our main contributions in this paper are as follows:

1. We are the first to advocate the use of Generative Adversarial Networks to enhance the details of positron images and realize the generation and processing of scarce industrial image data in the industrial non-destructive field.
2. We combine the attention-based mechanism in the professional domain image feature extraction. By constructing a memory module containing industrial positron image features, we can generate image generation in a specific domain, and conduct an industrial non-destructive positron image generative model finally.

## 2. Related Work

To improve the quality of reconstructed images, many methods of deep learning have been proposed in recent years. Ref. [10] proposed a multi-scale CNN approach based on the joint optimization of image content and texture constraints to get higher quality images. Ref. [11] trained a set of fast and effective convolutional neural network fusion modules based on prior knowledge to improve the image quality. Ref. [12] proposed to use the attention block to guide the convolutional neural network, which improves the image quality on the basis of reducing the network training complexity.

Nowadays, the GAN is one of the best deep learning methods in the field of image processing and achieves better performance. Refs. [13,14,15] used DCGAN to realize the batch generation of realistic medical images, and the resolution of the images passed” the Turing test” successfully. Ref. [16] used PGGAN to synthesize skin lesion images, and it proved to show highly realistic synthetic images successfully. Ref. [17] used CGAN to synthesize PET images by CT images and binary label graphs and proposed a multi-channel GAN to achieve a more realistic global output. Ref. [18] set up a multi-stage generator to get medical images under different conditions in turn by the intra-vascular ultrasound simulation of tissue maps according to different generative networks. Ref. [19] conducted joint learning by adding a specific task network to CGAN, then obtained a network model that retains specific task characteristics. Ref. [20] used WGAN as the network framework and uses noise and attribute vectors as inputs to generate high-resolution three-dimensional images. Ref. [21] combined the advantages of SRGAN [22] and RaGAN [23] and used residual dense block units and a relative average discriminator to make the edges of the reconstructed images sharper. Ref. [24] used the general reconstruction loss, gradient loss, and additional adversarial loss to train a full convolution network, and it successfully synthesized high quality real images. Ref. [25] proposed a solution focused on GAN for the augmentation of training data to improve the quality of MR images. Ref. [26] trained a GAN to generate synthetic MR images conditioned on various acquisition parameters and the Turing test proved the usefulness of generated images. Ref. [27] proposed a Tripartite Generative Adversarial Network with three associated networks to achieve CEMRI and the synthesized CEMRI had equivalent clinical value to real CEMRI.

The rest of the paper is organized as follows. The proposed method is described in Section 3. The experimental results and discussion are shown in Section 4. Finally, the conclusions are drawn in Section 5.

## 3. Methods

### 3.1. Encoder

The basic idea of the GAN comes from the zero-sum game theory. During the whole training, the two networks work against each other to get a good model. Mathematically, the model can be expressed as a “min-max” game in Equation (1):

(1) $$\underset{G}{min}\;\underset{D}{max}V\left(G,D\right)=\underset{G}{min}\;\underset{D}{max}E_{x\sim P_{data}}\left[logD\left(x\right)\right]+E_{z\sim P_{x}}\left[log\left(1-D\left(G\left(Z\right)\right)\right)\right]$$

where x represents real images, z represents the noise to the generator, G(Z) is the generated data, and D(x) is the probability of whether it is the real data.

Due to the uncontrollability of the initial model, we choose to add a prior to restricting the data generation, so that the generative model can be trained for industrial PET images.

Considering the scarcity of industrial positron images, we introduce the knowledge of migration learning and use medical images as training data to construct an encoder, which is based on the variational auto-encoder.

The specific implementation is as follows: we sample medical image data X to get a series of sample points $\left\{x_{1},x_{2},\;x_{3},\cdots x_{n}\right\}$, which makes all the sample data in X fit successfully and obtains a distribution p(x).

The distribution fitting of data sample X is finally realized with the help of implicit variable Z.

It is assumed that p(x) describes a probability distribution of X generated by Z and and it satisfies the Gauss distribution. Therefore, the whole encoder can be expressed as sampling Z from the standard normal distribution. In the process, we can get the variance and mean of sample data. The clustering process can be parameterized as Equation (2):

(2) $$\mu_{k}=f_{1}\left(X_{k}\right)\;log_{\sigma^{2}}=f_{2}\left(X_{k}\right)\;p\left(Z\right)=\sum_{X}p\left(Z|X\right)p\left(X\right)=\sum_{X}N\left(0,1\right)p\left(X\right)=N\left(0,1\right)$$

where the mean and variance of the normal distribution, which is exclusive to $X_{k}$, can be obtained. Then $Z_{k}$ can be sampled from this exclusive distribution.

### 3.2. Feature Extraction-Memory Module

After getting an image encoder, to obtain a more suitable generative model for PET images, we propose an image feature memory module based on the attention mechanism, which is used to extract domain image features.

The basic flow of the memory module is as follows: (1) use neural networks to extract the feature of the rare positron images and obtain the images’ feature vectors. (2) Combine the vector and the hidden variable in Section 3.2 based on attention mechanism to obtain an image memory model. (3) Use the memory model as the input of adversarial nets and train jointly with the whole network to obtain an industrial positron image generator

#### 3.2.1. Positron Image Feature Extraction

We use the principal component analysis [28] that is used to extract the positron sample data and the vector space transformation is used to reduce the dimensionality of higher dimensional positron data. Firstly, the original data are transformed into a new coordinate system by projection according to the new coordinate vector. Secondly, the variance of the first principal component of the projection data in the new coordinate system is the largest. As the dimension increases, the variance decreases in turn and the dimension decreases. It is described as Equation (3):

(3) $$Y=\left[\begin{array}{c} \begin{array}{c} y_{1}^{T}\\ y_{2}^{T} \end{array}\\ \begin{array}{c} \cdots \\ y_{m}^{T} \end{array} \end{array}\right]=\left[\begin{array}{cc} \begin{array}{c} \begin{array}{c} \begin{array}{cc} y_{1,1} & y_{1,2} \end{array}\\ \begin{array}{cc} y_{2,1} & y_{2,2} \end{array} \end{array}\\ \begin{array}{c} \begin{array}{cc} \cdots & \cdots \end{array}\\ \begin{array}{cc} y_{m,1} & y_{m,2} \end{array} \end{array} \end{array} & \begin{array}{c} \begin{array}{c} \begin{array}{cc} \cdots & y_{1,n} \end{array}\\ \begin{array}{cc} \cdots & y_{2,n} \end{array} \end{array}\\ \begin{array}{c} \begin{array}{cc} \cdots & \cdots \end{array}\\ \begin{array}{cc} \cdots & y_{m,n} \end{array} \end{array} \end{array} \end{array}\right]$$

m represents the positron sample, n represents the sample dimension and the sample Y=m×n.

The data matrix Y is de-averaged so that the mean value of each dimension is 0. Then, we find the most important feature vectors in the images, that is, the data on the coordinate axis represented by the feature fluctuates the most and the sum of squares of all samples projected on unit vector μ is the largest. Then we get the value of μ using Lagrange theorem. The mathematical expression is as Equation (4):

(4) $$u^{*}=argmax\frac{1}{m}\sum_{i=1}^{m}{\left(y_{i}^{T}u\right)}^{2}=argmaxu^{T}\left(\frac{1}{m}\sum_{i=1}^{m}y_{i}y_{i}^{T}\right)u^{N}\;L\left(\lambda ,u\right)=u^{T}\sum^{\;}u+\lambda \left(u^{T}u-1\right)\;\frac{\partial L\;}{\partial \mu }=\sum^{\;}u-\lambda u^{j}\;u^{*}=argmaxu^{T}\sum^{\;}u=argmax\lambda u^{T}u$$

The nets use convolution neural networks to construct an image feature extraction network, the network structure is divided into three layers, namely two convolution layers and one non-linear output layer. Firstly, small image slices are extracted from sample images and the dimension of the slices is the same as a convolution core. Then traverse all the pixels in them and perform a two-level convolution operation. Finally, hashing operation and histogram statistics are carried out in the output layer to print the feature vector.

#### 3.2.2. Memory Module Based on Attention Mechanism

The obtained positron eigenvector is fused with the hidden variable of the medical image obtained based on the attention mechanism to get the input nets. The purpose is to make the prior knowledge contained in the nets more focused on positron features so that the features of scarce data can be more applied in the whole training process.

The basic idea is the global attention and the focus in our model is to extract all positron image features. The specific realization is to align image data vectors, directly use positron images as query vectors, and input positron image feature vectors as a hidden state to calculate their weights, and the mathematical expression is shown in Equation (5):

(5) $$a_{t}\left(s\right)=align\left(z_{t},\bar{y_{s}}\right)=\frac{exp\left(score\left(z_{t},\bar{y_{s}}\right)\right)}{\sum_{s^{\prime }}exp\left(score\left(z_{t},{\bar{y}}_{st}\right)\right)}\;score\left(z_{t},\bar{y_{s}}\right)=z_{t}^{T}W_{a}\bar{y_{s}}$$

where $z_{t}$ is the medical image distribution, $\bar{y_{s}}$ are feature vectors extracted from positron images, and $score\left(z_{t},\;\bar{y_{s}}\right)$ is is the scoring criterion for the operation.

We get a constant and normalize it, and the contribution degree of each feature of the positron image to the network can be obtained. So, the image feature can be fused according to the weight ratio. Finally, the vector containing prior knowledge in the field is obtained as the overall input of adversarial nets.

### 3.3. Generative Adversarial Networks

#### 3.3.1. Generative Model

The generative network is constructed based on DenseNet [29], and the positron image features can be requisitioned repeatedly in the model. The network can also strengthen the contribution of the characteristics of scarce data so that the generated images are closer to real industrial positron images in detail.

The generative model is as follows: the output of the memory model in chapter 3.3 as a whole input to the net, and the input of each layer is related to the output of all the previous layers, not only related to an upper layer. It can be expressed as Equation (6):

(6) $$X_{l}=H_{l}\left(\left[X_{0},X_{1},\cdots ,X_{l-1}\right]\right)$$

$\left[X_{0},X_{1},\cdots ,X_{l-1}\right]$ is the concatenation to the net. We can group all output feature maps from layer $X_{0}$ to $X_{l-1}$ according to different channels and the structure is used to reduce the parameters without losing features randomly, so that the initial input can enter each layer’s convolution calculation to realize the feature reuse. The basic structure is a 3×3 convolution layer, Batch Normalization [30], and a ReLU non-linear activation layer.

Feature maps of all previous layers need to be a cat in the network. To perform the down sampling operation, the net is divided into several Dense blocks and transition layers are used between them. Referring to the original network, the net consists of Batch Normalization layers, a 1×1 convolution network, and a 2×2 average-pooling. In the same Dense block, the state of each layer is associated with all previous layers, and the training of each layer is aimed at the global state-feedback of the network to update the parameters.

#### 3.3.2. Discriminative Model

The discriminative net is used to discriminate specific images in a specific domain, in which domain image features can be used as the evaluation criteria for network classification as much as possible. The net uses the Markov Model based on PatchGAN, which is composed of full convolution layers. The output is an n-dimensional matrix. The mean of the matrix is used as the output of the discriminative network so that each receptive field in the image can be judged, which is equivalent to the convolution discriminant in batches by layers, and finally fed back to the whole network.

In the model, the real input samples are medical data. Therefore, in order to make the generated data better characterize positron image features, we need to add an additional attention perception loss function to the net. The loss function of the whole net consists of two parts: $L_{GAN}$ and $L_{APG}$. The loss function $L_{APG}$ is used to measure the distribution distance between the generated data and the positron images. The loss function is described as Equation (7):

(7) $$L_{APG}=E_{x,a\sim p\left(x,a\right)}\overset{s}{\underset{i=1}{\sum }}\frac{1}{W_{i}}\|D^{\prime }\left(x\right)-D\left(G\left(a\right)\right)\|$$

$W_{i}$ represents number of elements in each layer, and *s* is the number of layers. The loss function of the whole net can be described as Equation (8), and $L_{GAN}$ is similar as the original GAN.

(8) $$L_{GAN^{*}}=L_{GAN}+L_{APG}$$

### 3.4. Network Structure

**Figure 1:**
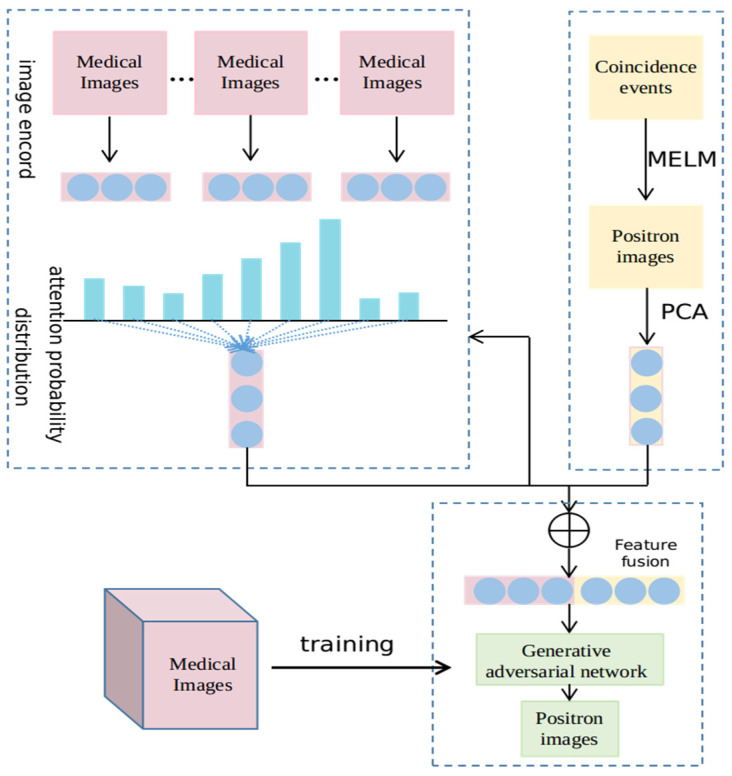
Network framework for generating positron images.

The overall view of the proposed network structure is shown in Figure 1. The basic framework is the generative adversarial nets and the input to the network consists of feature extraction and attention mechanism module.

Due to the limited number of positron images, research on positron images is few-shot learning. We have to extract common features of spectral images from other domains to enrich the encoding of the positron images for further study. We encode all spectral medical images to get their features from which the domain-specific feature can query common features of spectral images that are helpful for high image quality. We deem the encoded feature of positron images as the query and utilize the dot-product attention mechanism to retrieve common spectral features for the positron images and we enhance the positron image encoding by connecting the encoded domain-specific feature with its retrieval common feature.

The network is trained to obtain higher quality PET images and the experiment details are presented in the next section.

## 4. Experiments

### 4.1. Implementation Details

We design the model firstly by using an encoder to obtain the hidden vectors of the open-source medical image dataset and using principal component analysis to reduce positron images’ dimensionality and extract the main feature. Train memory module and adversarial nets jointly, and in the process of backpropagation, the identification network updates the parameters of the front-end network, so that the feature extraction network extracts the features repeatedly until the whole network achieves the optimal model. Finally, the positron image generator for industrial non-destructive testing is obtained.

The discriminator refers to the pixel and each batch is 70×70. The learning rate is 0.0002 in the whole net. The model is trained iteratively using Adam algorithm (β=0.5).

### 4.2. Experimental Data

The positron images are obtained by the Geant4 Application for Tomographic Emission (GATE). In the model design, we set some different templates with regular shapes based on the standardization of industrial parts. The relevant parameters are as follows: the anisotropic tube made of aluminum metal is filled with a positron nuclide solution; the activity is 600 Bq; the number of detectors is 184×64; the sampling time is 0.1 s; the energy resolution is 15%; the time resolution is 300 ps; the energy window is 350–650 keV; and the time window is 10 ns.

The design sampling time is 0.1 s to meet the needs of rapid sampling in the industrial field. Using the Maximum Likelihood-Expectation Maximization (MLEM) iteration algorithm to realize image preliminary reconstruction and obtain positron defect image in the industrial field.

### 4.3. Experimental Evaluations

We compare our approach with the commonly used generation model, aiming at the generation of industrial positron images. Here we use multi-scale structural similarity (MS-SSIM) [28] and the Peak Signal to Noise Ratio (PSNR) to measure the results of the experiment and the results are presented in Table 1.

By comparing the experimental data, we can see that the confrontation network constructed in this paper has a better effect on the generation of positron images for professional fields, and the generated images are closer to the real images.

**Figure 2:**
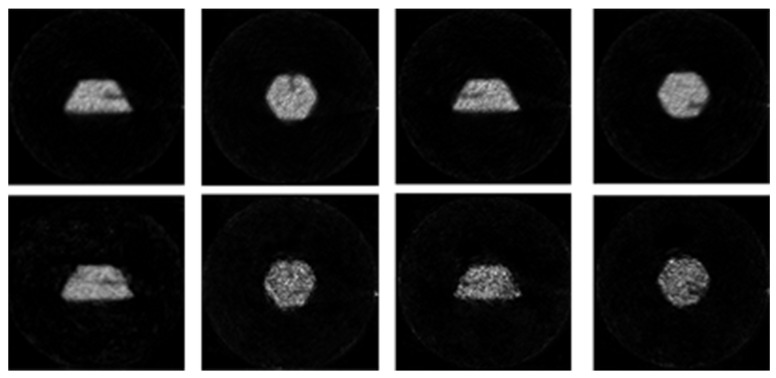
Comparison of positron images under different templates.

In addition, we process some industrial PET images by the method proposed in the paper. Some examples of pictures can be very clear. It can be seen clearly that the PET images have achieved good visual effects in Figure 2. The figure shows the imaging effects of different defects of industrial parts using our method. The second line is the original images and the first line is the processed images.

### 4.4. Experimental Discussions

**Figure 3:**
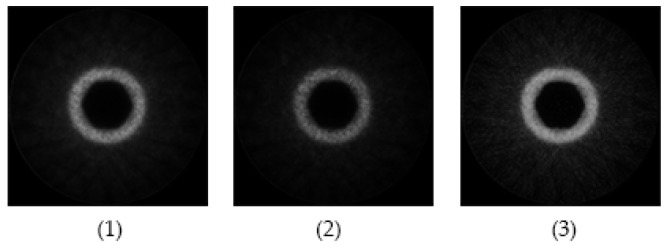
Image of hydraulic cylinder simulation data.

We conducted some experiments to prove the performance of the method in this article, mainly including (1) only generated by VAE or GAN; (2) generated by GAN with introducing attention mechanism; (3) mixed loss function is used based on model (2). Here, we selected relatively simple hydraulic cylinder simulation data for imaging, and the effect of imaging in different situations can be seen visually. The total activity is 1 mCi, 2.7 × 108 Bq, and the sampling time is 10 s. The imaging results are shown in Figure 3.

The three images in Figure 3 correspond to the imaging results under the above three conditions, and we can intuitively see that the third image has the best imaging effect.

**Figure 4:**
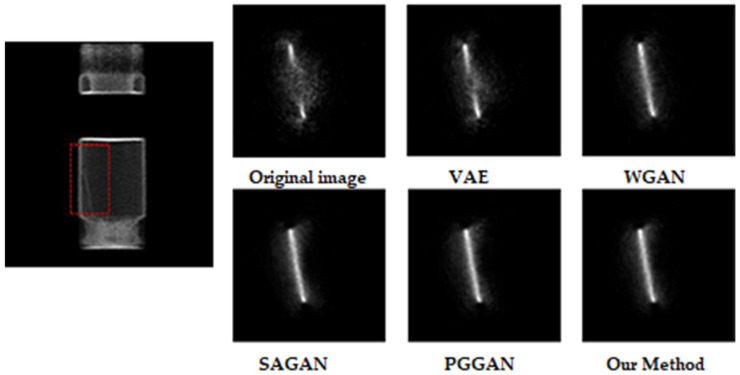
Experimental parameters: the concentration of nuclide is 800 Bq; the sampling time is 10 s; the material is the iron wire (foreign body) in the cavity.

Moreover, our application of PET technology in the field of industrial non-destructive testing is mainly focused on the gaps in complex cavities and the description of the internal flow field of industrial parts. Therefore, to further verify that the generative adversarial network based on the memory module constructed in this paper can obtain better image effects, we designed a group of experiments based on the industrial parts of the hydraulic cylinder. In the experiment, the PET detector we used was a Trans-PET-EXplorist 180, and the resolution of the detector crystal was 1 mm. Considering the actual size of the hydraulic parts, we injected about 350 mL of nuclide mixture with an activity configuration of 1.85 mCi. The shapes of foreign bodies in hydraulic parts under different models are shown in Figure 4.

We can see that the image quality obtained by the proposed method is the best from the figure, especially in the details of the image. In the practical application of industrial non-destructive testing, experts can better judge the internal conditions of the cavity based on the obtained images, so as to better realize the troubleshooting.

## 5. Conclusions

In this paper, we introduce an application of GAN in the field of nondestructive testing for specific industries. We combine the knowledge of transfer learning to make up for the problem of insufficient data. The key point is to introduce the attention mechanism to construct a positron image feature memory module, which can reuse image features under the condition of scarce data. At the same time, the attention loss function is added to the discriminative net to further improve the generator performance. Experiments show that compared with the state-of-the-art generation methods in deep learning, the model in our paper has an obvious improvement in the quality of industrial positron image generation.

In the future, our focus is to further study the application of generative adversarial networks in industrial positron image processing to further improve the quality of domain images.

## Figures and Tables

**Table 1:** The MS-SSIM and PSNR of different methods.

	PSNR	MS-SSIM
VAE	35.467	0.0485
WGAN	35.692	0.0567
SAGAN [31]	36.316	0.0598
PGGAN	36.677	0.0679
Our Method	36.913	0.0694

## References

[1] Goodfellow J., Pouget-Abadie M., Mirza B., Xu D., Warde-Farley S., Ozair A., Courville Y., Bengio Y. (2014). Advances in neural information processing systems. Curran Assoc..

[2] Isola P., Zhu J.-Y., Zhou T., Efros A.A., Isola P., Zhu J.Y., Zhou T. Image-to-Image Translation with Conditional Adversarial Networks. Proceedings of the IEEE Conference on Computer Vision and Pattern Recognition.

[3] Park S.J., Son H., Cho S. SRFeat: Single Image Super-Resolution with Feature Discrimination. Proceedings of the European Conference on Computer Vision (ECCV).

[4] Zhu J.Y., Park T., Isola P., Efros A.A. Unpaired Image-to-Image Translation using Cycle-Consistent Adversarial Networks. Proceedings of the IEEE International Conference on Computer Vision.

[5] Yu J., Lin Z., Yang J., Shen X., Lu X., Huang T.S. Generative Image Inpainting with Contextual Attention. Proceedings of the IEEE Conference on Computer Vision and Pattern Recognition.

[6] Radford A., Metz L., Chintala S. (2015). Unsupervised Representation Learning with Deep Convolutional Generative Adversarial Networks. Image and Graphics.

[7] Arjovsky M., Chintala S., Bottou L. (2017). Wasserstein GAN. Int. Conf. Mach. Learn..

[8] Mirza M., Osindero S. (2014). Conditional Generative Adversarial Nets. Comput. Sci..

[9] Karras T., Aila T., Laine S., Lehtinen J. (2017). Progressive Growing of GANs for Improved Quality, Stability, and Variation. arXiv.

[10] Zhang K., Zuo W., Gu S., Zhang L. Learning deep CNN denoiser prior for image restoration. Proceedings of the IEEE Conference on Computer Vision and Pattern Recognition.

[11] Tian C., Xu Y., Li Z., Zuo W., Fei L., Liu H. (2020). Attention-guided CNN for image denoising. Neural Netw..

[12] Chuquicusma M., Hussein S., Burt J., Bagci U. How to Fool Radiologists with Generative Adversarial Networks? A Visual Turing Test for Lung Cancer Diagnosis. Proceedings of the 2018 IEEE 15th International Symposium on Biomedical Imaging.

[13] Kitchen A., Seah J. (2017). Deep Generative Adversarial Neural Networks for Realistic Prostate Lesion MRI Synthesis. arXiv.

[14] Schlegl T., Seebck P., Waldstein S.M., Schmidt-Erfurth U., Langs G. Unsupervised Anomaly Detection with Generative Adversarial Networks to Guide Marker Discovery. Proceedings of the 2017 International Conference on Information Processing in Medical Imaging.

[15] Baur C., Albarqouni S., Navab N. (2018). Generating Highly Realistic Images of Skin Lesions with GANs. Proceedings of the International Conference on Medical Image Computing and Computer-Assisted Intervention.

[16] Wei W., Poirion E., Bodini B., Durrleman S., Ayache N., Stankoff B., Colliot O. (2018). Learning myelin content in multiple sclerosis from multimodal MRI through adversarial training. Proceedings of the International Conference on Medical Image Computing and Computer-Assisted Intervention.

[17] Tom F., Sheet D. Simulating Patho-realistic Ultrasound Images using Deep Generative Networks with Adversarial Learning. Proceedings of the 2018 IEEE 15th International Symposium on Biomedical Imaging (ISBI 2018).

[18] Bentaieb A., Hamarneh G. (2017). Adversarial Stain Transfer for Histopathology Image Analysis. IEEE Trans. Med. Imaging.

[19] Wolterink J.M., Leiner T., Isgum I. (2018). Blood Vessel Geometry Synthesis using Generative Adversarial Networks. arXiv.

[20] Wang X., Yu K., Wu S., Gu J., Lin Y., Dong C., Loy C.C., Qiao Y., Tang X. ESRGAN: Enhanced Super-Resolution Generative Adversarial Networks. Proceedings of the European Conference on Computer Vision (ECCV).

[21] Ledig C., Theis L., Huszar F., Caballero J., Cunningham A., Acosta A., Aitken A., Tejani A., Totz J., Wang Z. Photo-realistic single image super-resolution using a generative adversarial network. Proceedings of the IEEE Conference on Computer Vision and Pattern Recognition.

[22] Jolicoeur-Martineau A. (2018). The relativistic discriminator: A key element missing from standard GAN. arXiv.

[23] Dong N., Trullo R., Lian J., Petitjean C., Ruan S., Wang Q., Shen D. (2017). Medical Image Synthesis with Context-Aware Generative Adversarial Networks. Lecture Notes in Computer Science.

[24] Cui J., Li W., Gong W. (2020). Multi-stream attentive generative adversarial network for dynamic scene deblurring. Neurocomputing.

[25] Denck J., Guehring J., Maier A., Rothgang E. (2021). Enhanced Magnetic Resonance Image Synthesis with Contrast-Aware Generative Adversarial Networks. arXiv.

[26] Yang J., Dong X., Hu Y., Peng Q., Tao Q., Ou Y., Cai H., Yang X. (2020). Fully Automatic Arteriovenous Segmentation in Retinal Images via Topology-Aware Generative Adversarial Networks. Interdiscip Sci..

[27] Chan T.H., Jia K., Gao S., Lu J., Zeng Z., Ma Y. (2015). PCANet: A Simple Deep Learning Baseline for Image Classification?. IEEE Trans. Image Proc..

[28] Huang G., Liu Z., van der Matten L., Weinberger K.Q. Densely Connected Convolutional Networks. Proceedings of the IEEE Conference on Computer Vision and Pattern Recognition.

[29] Ioffe S., Szegedy C. Batch normalization: Accelerating deep network training by reducing internal covariate shift. Proceedings of the 32nd International Conference on Machine Learning.

[30] Odena A., Olah C., Shlens J. Conditional Image Synthesis with Auxiliary Classifier GANs. Proceedings of the 34th International Conference on Machine Learning.

[31] Zhang H., Goodfellow I., Metaxas D., Odena A. Self-Attention Generative Adversarial Networks. Proceedings of the 36th International Conference on Machine Learning.

